# Attitudes Towards Complementary and Alternative Medicine Among Pediatricians in Saudi Arabia

**DOI:** 10.7759/cureus.20486

**Published:** 2021-12-17

**Authors:** Alwaleed Alnafia, Faris H Binyousef, Abdulrahman Algwaiz, Anas Almazyed, Tariq Alduaylij, Osama Alolaiwi, Abdullah Alajlan, Mohammed Alsuhaibani, Kamel A Alenazi

**Affiliations:** 1 Medicine, Imam Mohammad Ibn Saud Islamic University, Riyadh, SAU; 2 Pediatrics, Qassim University, Qassim, SAU; 3 Pediatrics, Imam Mohammad Ibn Saud Islamic University, Riyadh, SAU

**Keywords:** saudi arabia, children, pediatricians, attitude, complementary and alternative medicine

## Abstract

Background

Complementary and Alternative Medicine (CAM) refers to a variety of healthcare practices outside the domain of conventional medicine, which may be integrated with conventional medicine by many physicians.

Objectives

This study aims to assess the attitudes and beliefs of pediatricians toward the use of CAM on children and to evaluate their knowledge and experience regarding CAM therapies and their desire for additional CAM training courses.

Methods

This is a cross-sectional study that was conducted in Saudi Arabia. A self-administered electronic questionnaire consisting of 27 questions was distributed among pediatricians to assess the demographics, beliefs, experiences, and attitudes of pediatricians related to the use of CAM for children. Non-probability convenience sampling was used in selecting the sample of pediatricians. Data analysis was performed using SPSS version 21 (IBM Corp., Armonk, NY).

Results

In this study, 140 participants completed the questionnaires. Most of the participants were of the male gender (83, 59.3%) and Saudi nationals (127, 90.7%). With regards to questions related to the experiences and opinions of participants regarding the use of CAM, 101 (72.1%) subjects reported the use of CAM among family members. Pediatricians cited affirmative encounters of use of CAM in 94 (67.1%) by parents. Pediatricians were not in favor of the use of CAM for end-stage (114, 81.4%) and chronic disease (108, 77.1%). The overall median self-reported CAM knowledge score was statistically higher for males in comparison with females [3 (IQR 1-5)] versus 2 (IQR 2-4), *P* = 0.030]. Also for nationality, median scores were significantly greater for the Riyadh region in contrast to other regions [[3 (IQR 2-5)] versus 2 (IQR 1-4), *P* = 0.041]. There was a significant difference in median scores for qualification of participants (*P* = 0.002). A multiple pairwise comparison revealed a statistically significant (*P* = 0.012) difference between participants with the qualification of residents and specialization. No differences in median CAM scores were found for responses related to the experiences, opinions, and attitudes of pediatricians towards the use of CAM therapies.

Conclusion

Most of the pediatricians in Saudi Arabia don’t recommend the use of CAM treatment in their practice, but they acknowledge that learning more about CAM and imparting appropriate knowledge regarding it may incorporate its use in their routine clinical practices in a safe way.

## Introduction

Complementary and Alternative Medicine (CAM) can be defined as a healthcare domain that refers to an array of diagnostic and treatment approaches unlike conventional medicine and whose core principles every so often do not comply with existing healthcare principles and guidelines [1-2]. The practice of CAM is gaining traction lately, especially in Western nations. According to one report, around three-quarters (74%) of Canadians have utilized traditional, complementary, alternative, and integrative medicine [3]. The forms of CAM used are widely different among the populations. For example, herbal remedies, acupuncture, therapeutic massage, special exercises, homeopathy, phototherapy, traditional Chinese medicine, nutritional supplements, and megadose vitamins are commonly used in American and European countries [4-6]. In Saudi Arabia, the most common forms used are prayers, the Holy Quran recitation, Zamzam water, cupping (Hijama), acupuncture, Nigella sativa (black seed), herbs, honey and other dietary products, and camel milk and urine [7-8].

In Saudi Arabia, the use of CAM ranges from 50% to 70%, depending on the specific regions [9]. Parents frequently seek CAM that is consistent with their cultural beliefs in order to avoid conventional treatment and believe that it will help their child feel better [10]. Although the majority of parents prefer to discuss the use of CAM with their pediatricians, only 36% of them do so [11]. For valid reasons of safety and effectiveness of CAM, communication between pediatricians and parents is vital, and conversely, a progressive inter-consultation decline has been witnessed regarding the discussion of the use of CAM [12]. Therefore, pediatricians are routinely encouraged to offer parents unbiased guidance on the use of CAM as a therapeutic modality [13]. For well-poised CAM-related guidance, adequate knowledge and open-mindedness towards CAM are essential.

The aim of the present study was to determine the pediatricians’ attitudes and beliefs toward the use of CAM on children, evaluate their knowledge and experience regarding CAM therapies, and finally, assess their desire and experience for additional CAM training courses. The findings of the present study may encourage the development of CAM medical education programs and physicians to discuss the use of CAM with parents in order to plug the communication gap and debunk associated myths. This will consequently promote reliable and safer clinical practice.

## Materials and methods

A descriptive cross-sectional, questionnaire-based study was performed in Saudi Arabia. The study underwent review and ethical approval was obtained from the ethical review committee of the College of Medicine, Imam Muhammad Ibn Saud Islamic University, Riyadh, Saudi Arabia.

The study sample comprised Saudi and non-Saudi pediatricians working in Saudi Arabia. Non-probability convenience sampling was used in selecting the pediatricians. The questionnaire was developed on the Google Forms platform to acquire the data related to study objectives. The link of the questionnaire was sent to 750 different pediatricians in Saudi Arabia via e-mails and WhatsApp messages, and we have received 140 responses. The main reason for sending out questionnaires via e-mail and WhatsApp messages was to collect greater participation responses from diverse sociodemographic backgrounds and to recognize the ease of participation for pediatricians. The progressive increase of response on the Excel sheet (Microsoft Corporation, Redmond, WA) was considered as the disposition to participate.

An online structured pilot-tested questionnaire was developed and comprised a total of 27 questions. Six questions recorded the sociodemographic characteristics of the participating pediatricians, 12 questions assessed the experiences and opinions of pediatricians towards the use of CAM, two questions documented the self-reported CAM knowledge scores and source of CAM knowledge, and the final seven questions determined the attitude of pediatricians regarding the utilization of CAM (5-point Likert scale responses: “Agree”, “Somewhat Agree”, “Neutral”, “Somewhat Disagree", and “Disagree”).

The data was transferred from the Excel sheet to the Statistical Packages for Social Sciences (SPSS) software version 21 (IBM Corp., Armonk, NY). The data entry, cleaning, and analyses were performed using SPSS. Descriptive analyses were executed. Continuous data were presented as mean ± standard deviation (SD) or median and interquartile range (IQR) and categorical data as frequencies and percentages. The P-value significance for all tests was set at <0.05. The Mann-Whitney U or Kruskal-Wallis test, when appropriate, was used to compare the self-reported CAM knowledge scores and demographic characteristics.

## Results

In the present study, 140 participants completed and returned the questionnaires. Most of the participants were of the male gender (83, 59.3%) and Saudi nationals (127, 90.7%). Distribution of participants based on age groups was as follows: 21-30 (54, 38.6%), 31-40 (63, 45%), 41-50 (17, 12.1%), 51-60 (5, 3.6%), and 60 above (1, 0.7%). A large number of participants were from the Riyadh region (76, 54.3%). Fifty-nine (42.1%) participants were consultants, followed by 42 (30%) on-training residents. The specialty of general pediatrics (74, 52.9%) was most prominent in our cohort. Table 1 shows the socioeconomic features of the participants.

**Table 1:** Sociodemographic characteristics of study subjects (n = 140)

­	n (%)
Gender	
Males	83 (59.3)
Females	57 (40.7)
Age Groups in Years	
21-30	54 (38.6)
31-40	63 (45.0)
41-50	17 (12.1)
51-60	5 (3.6)
60 above	1 (0.7)
Nationality	
Saudi	127 (90.7)
Non-Saudi	13 (9.3)
Region	
Al-Madinah	4 (2.9)
Aseer	13 (9.3)
Eastern Province	11 (7.9)
Hail	5 (3.6)
Jazan	8 (5.7)
Makkah	15 (10.7)
Northern Borders Province	2 (1.4)
Qassim	4 (2.9)
Riyadh	76 (54.3)
Qualification	
Resident (on training)	42 (30.0)
Resident (not on training)	4 (2.9)
Fellow	16 (11.4)
Consultant	59 (42.1)
Specialist	19 (13.6)
Specialization	
Allergy/Immunology	3 (2.1)
Endocrinology	8 (5.7)
Emergency	2 (1.4)
Gastroenterology	7 (5.0)
General Pediatrics	74 (52.9)
Genetics/Metabolic Diseases	2 (1.4)
Hemato-Oncology	5 (3.6)
Infectious Disease	3 (2.1)
Neonatal ICU	13 (9.3)
Nephrology	1 (0.7)
Neurology	6 (4.3)
Palliative Care	2 (1.4)
Pediatric Emergency	3 (2.1)
Pediatric ICU	7 (5.0)
Pulmonology	3 (2.1)
Rheumatology	1 (0.7)

With regards to questions related to the experiences and opinions of participants regarding the use of CAM, 101 (72.1%) subjects reported the use of CAM among family members. The data suggested that parents frequently (42, 30%) ask pediatricians about CAM therapy. Furthermore, participants cited affirmative encounters of the use of CAM in 94 (67.1%) by parents. When asked about their opinion on suggesting the use of CAM for the pediatric population with end-stage or chronic disease, 114 (81.4%) and 108 (77.1%) of the participants responded “No,” respectively. However, a large number of subjects were equally positive (62, 44.3%) and uncertain (62, 44.3%) about the importance of learning CAM for pediatricians. Table 2 depicts the experiences and opinions of pediatricians towards the use of CAM. When asked about the specific CAM therapies that their family members used for treatment, the participants reported asafoetida, Astragalus sarcocolla, anise, cumin, myrrh, fenugreek, Nigella sativa, oils, and peppergrass to be in frequent use. Similar CAM therapies were also considered safe by pediatricians in children.

**Table 2:** Experiences and opinions of pediatricians towards the use of CAM Abbreviation: CAM, Complementary and Integrative Medicine

Variables	n (%)
Did you or your close family members (children, spouse, and parents) use Traditional Medicine at any time?	
Yes	101 (72.1)
No	39 (27.9)
How often do patient parents ask you about traditional/complementary medicine?	
Never	3 (2.1)
Rarely	11 (7.9)
Often	35 (25.0)
Sometimes	38 (27.1)
Usually	42 (30.0)
Always	11 (7.9)
Did you encounter a patient who received traditional treatment for his medical problem this year?	
Yes	94 (67.1)
No	46 (32.9)
Would you suggest Traditional Medicine to patients, if the patient has end-stage cancer or other medical conditions without a cure?	
Yes	26 (18.6)
No	114 (77.1)
Would you suggest Traditional Medicine to patients, if the patient has a chronic illness that cannot be completely treated by conventional Modern Medicine?	
Yes	32 (22.9)
No	108 (77.1)
Do you think learning Traditional Medicine is important for pediatricians?	
Yes	62 (44.3)
No	16 (11.4)
May be	62 (44.3)

**Figure 1:**
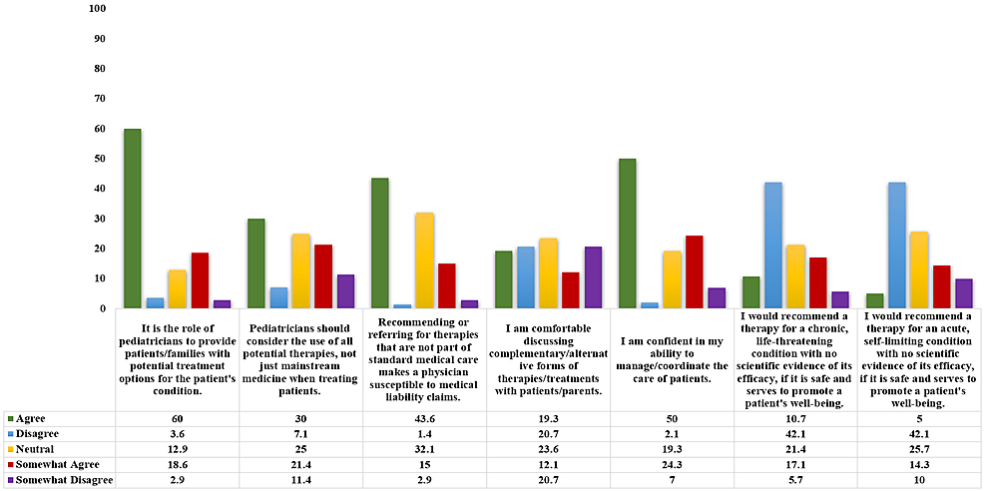
Attitudes of pediatricians towards the use of CAM Percentages of all the responses (agree, disagree, neutral, somewhat agree, and somewhat disagree) are shown. Abbreviation: CAM, Complementary and Integrative Medicine

Figure 1 demonstrates the attitudes of pediatricians towards the use of CAM. Pediatricians agreed that they should provide parents with every treatment option available (60%) and consider potential, available therapies (30%). However, participants also agreed that recommending CAM therapies would make them susceptible to liability claims (43.6%). Nearly one-fourth (23.6%) of the participants were neutral with regards to the comfortability of discussing the CAM treatment options with the parents. The majority of the participants categorically disagreed to use CAM with no evidence even if it serves the purpose of benefitting the patient with acute or self-limiting (42.1%) and chronic illness (42.1%).

The overall median self-reported CAM knowledge score was statistically higher for males in comparison with females [3 (IQR 1-5)] versus 2 (IQR 2-4), P = 0.030]. When compared for nationality, median scores were significantly greater for the Riyadh region in contrast to other regions [[3 (IQR 2-5)] versus 2 (IQR 1-4), P = 0.041] in Saudi Arabia altogether (Table 3). Significant differences in median CAM knowledge scores were also found for age groups (P = 0.017); however, when pairwise comparison was executed, no differences were observed (P ≥ 0.05) (Tables 4-5). There was a significant difference in median scores for qualification of participants (P = 0.002). The multiple pairwise comparison revealed a statistically significant (P = 0.012) difference between participants with the qualification of residents and specialization (Tables 6-7).

**Table 3:** Comparison of self-reported CAM knowledge scores based on gender, nationality, and region of participants (Mann-Whitney U test) Abbreviation: CAM, Complementary and Integrative Medicine Bold P-values are statistically significant. *Due to less number of participants, regions other than Riyadh were merged for analysis.

Variables	Median and IQR of CAM Knowledge Score (Out of 10)	P-Value
Self-reported CAM knowledge scores and gender		
Males	3 (1-5)	0.030
Females	2 (1-4)	
Self-reported CAM knowledge scores and nationality		
Saudi	3 (1-5)	0.133
Non-Saudi	4 (2.5-5)	
Self-reported CAM knowledge scores and region		
Riyadh	3 (2-5)	0.041
Other regions*	2 (1-4)	

**Table 4:** Comparison of self-reported CAM knowledge scores based on age groups of participants (Kruskal-Wallis test) Abbreviation: CAM, Complementary and Integrative Medicine The bold P-value is statistically significant.

Variables	Median and IQR of CAM Knowledge Score (Out of 10)	P-Value
21-30	2 (1-4)	0.017
31-40	3 (1-5)	
41-50	3 (2.5-6.5)	
51-60	4 (3-6)	
60 above	-	

**Table 5:** Comparison of self-reported CAM knowledge scores based on age groups of participants (Kruskal-Wallis pairwise comparison test) Abbreviation: CAM, Complementary and Integrative Medicine The bold P-value is statistically significant. *Bonferroni Adjusted P-Values

Variables	P-Value*
21-30 vs. 31-40	1.000
21-30 vs. 41-50	0.077
21-30 vs. 51-60	0.450
21-30 vs. 60 above	0.851
31-40 vs. 41-50	0.800
31-40 vs. 51-60	1.000
31-40 vs. 60 above	1.000
41-50 vs. 51-60	1.000
41-50 vs. 60 above	1.000
51-60 vs. 60 above	1.000

**Table 6:** Comparison of self-reported CAM knowledge scores based on the qualification of participants (Kruskal-Wallis test) Abbreviation: CAM, Complementary and Integrative Medicine The bold P-value is statistically significant.

Variables	Median and IQR of CAM Knowledge Score (Out of 10)	P-Value
Resident (on training)	2 (1-4)	0.002
Resident (not on training)	0.5 (0-1)	
Fellow	2 (1.25-4)	
Consultant	4 (2-5)	
Specialist	3 (1-5)	

**Table 7:** Multiple comparisons of self-reported CAM knowledge scores based on the qualification of participants (Kruskal-Wallis pairwise comparison test) Abbreviation: CAM, Complementary and Integrative Medicine The bold P-value is statistically significant. *Bonferroni adjusted P-values

Variables	P-Value*
Resident (on training) vs. Resident (not on training)	0.595
Resident (on training) vs. Fellow	1.000
Resident (on training) vs. Consultant	0.051
Resident (on training) vs. Specialist	1.000
Resident (not on training) vs. Fellow	0.335
Resident (not on training) vs. Consultant	0.131
Resident (not on training) vs. Specialist	0.012
Fellow vs. Consultant	0.267
Fellow vs. Specialist	1.000
Consultant vs. Specialist	1.000

Finally, we also analyzed median CAM knowledge scores for differences against experiences, opinions, and attitudes of pediatricians towards the use of CAM therapies (Table 8). We witnessed no statistical difference between median CAM knowledge scores and following response questions: use of CAM in acquaintance (P = 0.764), experience of parents asking about CAM therapy (P = 0.161), attending patient with recent history of CAM use (P = 0.611), opinion regarding suggestion of CAM use in end-stage disease (P = 0.219) and chronic illness (P = 0.059), importance of learning CAM for pediatricians (P = 0.813), perception regarding the role of pediatricians to provide patients/families with information about all potential treatment options for the patient's condition (P = 0.805), considerations of pediatricians for the use of all potential therapies, not just those of mainstream medicine when treating patients (P = 0.456), perception regarding physician’s susceptibility to medical liability on recommendation or referral for therapies that are not part of standard medical care (P = 0.140), comfortability of pediatricians in discussing CAM therapies/treatments with patients/parents (P = 0.475), confidence of pediatricians in the ability to manage/coordinate the care of patients (P = 0.071), suggestion of the use of CAM for patients with chronic disease (P = 0.491), and perception regarding recommendations for the use of CAM for an acute, self-limiting condition with no scientific evidence of its efficacy if it is safe and serves to promote a patient's well-being (P = 0.647).

**Table 8:** Mean difference of self-reported CAM knowledge scores in relation to the experiences, opinions, and attitudes of pediatricians towards the use of CAM (Kruskal-Wallis test) Abbreviation: CAM, Complementary and Integrative Medicine; ANOVA, Analysis of Variance; IQR, Interquartile Range

Variables	Median and IQR of CAM Knowledge Score (Out of 10)	P-Value
Self-reported CAM knowledge scores and use of CAM in acquaintance		
Yes	3 (1-5)	0.764
No	3 (2-5)	
Self-reported CAM knowledge scores and experience of patient’s parents asking about CAM		
Never	1 (1-0)	0.161
Rarely	2 (1-3)	
Often	3 (2-5)	
Sometimes	3 (1-5)	
Usually	2 (1-4)	
Always	2 (2-6)	
Self-reported CAM knowledge scores and attending patient with a history of use of CAM in 2021		
Yes	3 (2-5)	0.611
No	2 (1-5)	
Self-reported CAM knowledge scores and opinion regarding the suggestion of the use of CAM for patients with chronic illness		
Yes	3 (1-5)	0.219
No	3 (1-5)	
Self-reported CAM knowledge scores and opinion regarding the suggestion of the use of CAM for patients with acute illness		
Yes	3 (2-5)	0.059
No	2 (1-5)	
Self-reported CAM knowledge scores and opinions regarding the importance of learning CAM for pediatricians		
Yes	2.5 (1-5)	0.813
No	3 (2-4.75)	
May be	3 (1-5)	
Self-reported CAM knowledge scores and perception regarding the role of pediatricians to provide patients/families with information about all potential treatment options for the patient's condition		
Agree	3 (2-5)	0.805
Disagree	3 (2-6)	
Neutral	2.5 (1.75-5)	
Somewhat Agree	2.5 (1-5)	
Somewhat Disagree	2.5 (1.25-3.75)	
Self-reported CAM knowledge scores and considerations of pediatricians for use of all potential therapies, not just those of mainstream medicine when treating patients		
Agree	2.5 (1-4.25)	0.456
Disagree	3.5 (1.75-5.25)	
Neutral	4 (1.5-5)	
Somewhat Agree	3 (2-5)	
Somewhat Disagree	2 (1-3)	
Self-reported CAM knowledge scores and perception regarding physician’s susceptibility to medical liability on recommendation or referral for therapies that are not part of standard medical care		
Agree	3 (2-5)	0.140
Disagree	5 (4-0)	
Neutral	3 (2-5)	
Somewhat Agree	2 (1-4)	
Somewhat Disagree	2 (0.5-5.75)	
Self-reported CAM knowledge scores and comfortability of pediatricians in discussing CAM therapies/treatments with patients/parents		
Agree	2.5 (1-4.25)	0.475
Disagree	3 (2-5)	
Neutral	4 (2-5)	
Somewhat Agree	2.5 (1.25-3.75)	
Somewhat Disagree	2.5 (1-4.75)	
Self-reported CAM knowledge scores and confidence of pediatricians in the ability to manage/coordinate the care of patients		
Agree	3 (1-4)	0.071
Disagree	6 (2-0)	
Neutral	4 (2-5)	
Somewhat Agree	2 (1-4.75)	
Somewhat Disagree	-	
Self-reported CAM knowledge scores and suggestions of the use of CAM for patients with chronic disease		
Agree	2 (1-4.25)	0.491
Disagree	3 (2-5)	
Neutral	3 (2-5)	
Somewhat Agree	4 (1-5)	
Somewhat Disagree	1.5 (0.25-4.75)	
Self-reported CAM knowledge scores and perception regarding recommendations for the use of CAM for an acute, self-limiting condition with no scientific evidence of its efficacy, if it is safe and serves to promote a patient's well-being		
Agree	3 (0.75-5.5)	0.647
Disagree	3 (1.5-4)	
Neutral	4 (2-5)	
Somewhat Agree	2 (1-4)	
Somewhat Disagree	3 (0.75-5)	

## Discussion

The term CAM refers to a variety of therapies that are prevalent in both healthy people and those with clinical conditions [14]. Complementary therapies have been widely used in the treatment of many chronic diseases, especially in the West. For example, the most common medical practices in Europe are acupuncture, homeopathy, manual therapy or manipulation, simple therapy, or herbal medicine [15-16]. A systematic review was conducted to review studies on CAM use in Saudi Arabia; they found that the most common form was prayer and the recitation of the Holy Quran, followed by herbs, honey, food products, and then cupping therapy [8]. CAM-related office physicians' opinions and behaviors have been tested in a number of surveys, and physicians have shown great interest in CAM. Lack of physician-patient contact regarding CAM may reduce the chances of discussing the benefits of CAM and the differences between medications and CAM treatments [17]. However, as far as we know, there are no studies in which pediatricians in Saudi Arabia have an opinion on the use of CAM, therefore, the purpose of this study was to evaluate the attitudes and beliefs of pediatricians towards using CAM on children.

The present online questionnaire-based survey among pediatricians in Saudi Arabia found that a large number of Saudi pediatricians have a slightly negative attitude towards the use of CAM. Despite nearly half of the participants acknowledging the importance of learning CAM for pediatricians and the frequent use of CAM by Saudi parents [4,18], a relatively high number of pediatricians were not in favor of using CAM for the pediatric population with end-stage or chronic disease. This finding is interesting for two reasons: 1) as reported in the literature, the use of CAM by parents is mostly driven by factors such as children’s age less than one year, low parent education, neurological or chronic disease, and a family history of CAM use and religion [4,18], which may not be the case with pediatricians because their routine practice is largely based on scientific evidence, and 2) another possible reason could be the knowledge deficit regarding CAM; pediatricians may have little knowledge, which is gathered through their own family experience or by encountering a patient narrating the use of CAM.

The study demonstrated that 30% of the Saudi parents asked pediatricians about CAM. These findings are in agreement with an earlier report; most of the time, the discussion of using CAM treatment starts on the part of parents [19]. One study from the Netherlands also reported that 60% of the parents expressed their intention to discuss with pediatricians about earlier or imminent utilization of CAM [13]. Therefore, from the parent's standpoint, it is suggested that pediatricians should routinely question CAM therapy during consultations. The American Academy of Pediatrics (AAP), in 2009, issued a list of communication techniques that assist pediatricians to discuss CAM with parents [20]. The paper included techniques like questioning about types of treatments being used by parents, keeping in mind their value and belief systems, working together through active listening of parents. These strategies laid by the AAP are pertinent to Saudi pediatricians, as the use of CAM is very rampant among Saudi parents [4,18].

Despite pediatricians encountering a high use of CAM in terms of family members and parents, one factor that may deter pediatricians to discuss CAM with patients could be a knowledge deficit and lack of training in CAM. At present, there are no formal training programs regarding CAM in Saudi Arabia. The soaring prevalence of CAM use demonstrates a dire need to develop and promote a formal course on CAM, which should be a permanent part of medical education in Saudi Arabia. In the present, 44.3% of the participants also acknowledged the importance of learning CAM for pediatricians.

Earlier studies have shown that pediatricians are likely to recommend CAM for children with chronic issues like abdominal pain, headaches, asthma, chronic pain, mental health, and psychiatric problems, when conventional medicine fails [21-23]. Moreover, a study conducted by Sikand and Laken indicated that 45.8% of the pediatricians would use CAM for their patients in case of failure of traditional treatment and 14.8% for untreatable chronic conditions [24]. However, this study showed that pediatricians were not in favor of recommending CAM therapy for patients with acute and chronic health care conditions. The reason for this discrepancy could be a lack of knowledge and therefore reluctance to refer CAM therapy.

We found that a higher level of knowledge toward using CAM was noticed in male physicians, older participants, and consultants. Furthermore, we found a significantly lower level of knowledge of CAM among residents, especially those without training. In a study by Marie et al., the authors found that most of the participants had poor knowledge of CAM with no significant difference between specialists; however, physicians of Saudi origin tend to believe that CAM is beneficial; those of non-Saudi origin tended to have a more negative attitude. Moreover, the study found physicians employed as residents, and those new to practicing medicine, were more positive towards CAM [25]. Moreover, another study did find any difference between genders or age in knowledge or attitude toward using CAM [10].

The present study contributes to the growing body of knowledge regarding the beliefs and attitudes of Saudi pediatricians towards the use of CAM and in advocating the knowledge dissemination of CAM in Saudi Arabia. It is important to note that 44.3% of pediatricians believe that learning CAM is of significance to pediatricians. While numbers may be low for pediatricians in favor of using CAM for children at the moment, but appropriately educating them might shift the dynamics and acceptability of the use of CAM in modern medicine. There is a need for large-scale research studies to unearth the role, effectiveness, and safety profile of CAM among the pediatric population. It was not astonishing that in the present study, Saudi pediatricians were concerned not only about the side effects but also about the fact that there is no evidence of effectiveness and drug interactions related to the use of CAM therapies. Our findings are in line with that of the USA, where most of the pediatricians (75%) are concerned about the safety profile of CAM therapies and that it may bring added side effects [26].

The present study has a few limitations. First, the sample size is too small to generalize study findings and therefore a large study needs to be conducted. However, the study enrolled participants from different regions of Saudi Arabia. Another limitation of this study is that the study is questionnaire-based and distributed online. This might lead to a response bias on part of the participants. Furthermore, the present study relied on self-reported rather than observational measures of the pediatricians’ practice. Hence, the reported findings might avert from what we observe in the routine practice of pediatricians. Finally, beliefs and attitudes towards the recommendation of CAM therapies might have been different if participants were questioned about other basic clinical conditions like intractable abdominal pain where every so often routine medical care does not add to patient recovery and pediatricians might be more open to considering CAM therapies. This is what we witnessed in the present study; in addition, most pediatricians believed that CAM therapy may help in alleviating gastric and respiratory issues in children.

## Conclusions

In conclusion, a large number of Saudi pediatricians have a negative response towards the use and recommendation of CAM treatment. Nevertheless, pediatricians understand the importance of learning CAM, and imparting appropriate knowledge about CAM may lead to the incorporation of CAM use in their routine clinical practices.

## Appendices

Questionnaire

1. Gender: Male / Female

2. Age (years): 21-30 / 31-40 / 41-50 / 51-60 / 60 above

3. Nationality Saudi/Non-Saudi

4. Region:

5. Qualification: Resident in training / Resident not in training / Fellow / consultant

6. Specialization:

7. Did you or your close family members (children, spouse, and parents) use Traditional Medicine at any time? Yes / No

8. If yes, what do you or your family use/ed? (You may select more than one option)

o   Myrrh

o   Asafoetida

o   Nigella sativa

o   Oils

o   Cumin

o   Anise

o   peppergrass

o   Astragalus sarcocolla

o   Fenugreek

o   /Geum urbanum

o   Alkanna tinctoria

o   Others:

9. How often do parents ask you about traditional/complementary medicine: Always / usually / often / sometimes / rarely / never

10. Did you encounter a patient who received traditional treatment for his medical problem this year? Yes / No

11. If yes, how many patients? 1-3 / 4-7 / 8-11 / 11- 14 / 15 or more

12. Traditional therapies which you consider to be safe in the pediatric population: (select all that apply)

o   Myrrh

o   Asafoetida

o   Nigella sativa

o   Oils

o   Cumin

o   Anise

o   peppergrass

o   Astragalus sarcocolla

o   Fenugreek

o   /Geum urbanum

o   Alkanna tinctoria

o   Others:

13. Would you suggest Traditional Medicine to patients, if the patient has end-stage cancer or other medical conditions without a cure: Yes / No

14. Would you suggest Traditional Medicine to patients, if the patient has a chronic illness that cannot be completely treated by conventional Modern medicine? Yes / No

15. What makes you concerned about patients using Traditional Medicine (select all that apply): No evidence of effectiveness / drug interactions / side effects / more reliance over evidence-based medicine

16. In which of the following conditions would you see a role of Traditional Medicine in alleviating the child's medical condition? (You may select more than one option)

O Asthma

O Hematological diseases (e.g., Sickle cell disease, thalassemia)

O Joint problems (e.g., arthritis, backaches)

O Neurological disorders (e.g., cerebral palsy)

O Nausea and vomiting

O Cancer

O Digestive tract problems

O Diabetes mellitus

O Respiratory tract infection

O Strengthen the immune system

O Skin disorders (including eczema)

O General pain management

O Behavioral problems (e.g., attention-deficit hyperactive disorder, nightmares, bedwetting, eating disorders)

O Headaches

O Others:

17. How would you rate your knowledge of Traditional Medicine on a scale of 0 to 10? (0 No knowledge, 10 Excellent knowledge)  0 / 1 / 2 / 3 / 4 / 5 / 6 / 7 / 8 / 9 / 10

18. Where did you acquire your Knowledge of Traditional Medicine (if applicable)? Traditional Medicine degree / training course / newspapers / patients / relatives / traditional practitioners / other media

19. Do you think learning Traditional Medicine is important for pediatricians? Yes / No

20. If yes, what is the reason? (You may select more than one option)

O To answer the parent's questions related to Traditional Medicine

O To counsel parents to use Traditional Medicine

O To counsel parents to avoid Traditional Medicine

O Other:

21. It is the role of pediatricians to provide patients/families with information about all potential treatment options for the patient's condition? Agree - Somewhat agree - Neutral - Somewhat disagree - Disagree

22. Pediatricians should consider the use of all potential therapies, not just those of mainstream medicine when treating patients? Agree - Somewhat agree - Neutral - Somewhat disagree - Disagree

23. Recommending or referring for therapies that are not part of standard medical care makes a physician susceptible to medical liability claims? Agree - Somewhat agree - Neutral - Somewhat disagree - Disagree

24. I am comfortable discussing complementary/alternative forms of therapies/treatments with patients/parents? Agree - Somewhat agree - Neutral -Somewhat disagree - Disagree

25. I am confident in my ability to manage/coordinate the care of patients? Agree - Somewhat agree - Neutral -Somewhat disagree - Disagree

26. I would recommend a therapy for a chronic, life-threatening condition with no scientific evidence of its efficacy, if it is safe, and serves to promote a patient's well-being? Agree - Somewhat agree - Neutral - Somewhat disagree - Disagree

27. I would recommend a therapy for an acute, self-limiting condition with no scientific evidence of its efficacy, if it is safe, and serves to promote a patient's well-being? Agree - Somewhat agree - Neutral - Somewhat disagree - Disagree

## References

[1] Dhankar M (2018). Complementary and alternative medicine: a cross-sectional observational study in pediatric inpatients. J Evid Based Integr Med.

[2] Huber BM, von Schoen-Angerer T, Hasselmann O, Wildhaber J, Wolf U (2019). Swiss paediatrician survey on complementary medicine. Swiss Med Wkly.

[3] Esmail N (2007). Complementary and alternative medicine in Canada: trends in use and public attitudes, 1997-2006. Public Policy Sources.

[4] Al-Rumayyan A, Alqarni H, Almanna BS, Althonayan N, Alhalafi M, Alomary N (2020). Utilization of complementary medicine by pediatric neurology patients and their families in Saudi Arabia. Cureus.

[5] Wilson KM, Klein JD (2002). Adolescents' use of complementary and alternative medicine. Ambul Pediatr.

[6] Sawni-Sikand A, Schubiner H, Thomas RL (2002). Use of complementary/alternative therapies among children in primary care pediatrics. Ambul Pediatr.

[7] AlShehri SD, AbdulHameed RM, Taha AZ, Almusalmi AM, Almulaify MS, Alkhabbaz FL (2020). Complementary and alternative medicine practice and perceptions of attendees of primary care centers in Eastern Saudi Arabia. J Family Community Med.

[8] Alrowais NA, Alyousefi NA (2017). The prevalence extent of Complementary and Alternative Medicine (CAM) use among Saudis. Saudi Pharm J.

[9] Al-Bedah A, Qureshi N, Al-Yahia O (2017). Current status of traditional and complementary medicine use in Qassim Province, Saudi Arabia. Journal of Complementary and Alternative Medical Research.

[10] Al Saadoon MA, Al Jafari MS, Al Dhouyani BD, Rizvi S (2015). Factors associated with pediatrician attitudes over the use of complementary and traditional medicine on children in Muscat, Oman. Int J Health Policy Manag.

[11] Sibinga EM, Ottolini MC, Duggan AK, Wilson MH (2004). Parent-pediatrician communication about complementary and alternative medicine use for children. Clin Pediatr (Phila).

[12] Abdullah Al-Rowais N, Al Bedah AM, Khalil MK (2012). Knowledge and attitudes of primary health care physicians towards complementary and alternative medicine in the Riyadh region, Saudi Arabia. Forsch Komplementmed.

[13] Vlieger AM, van Vliet M, Jong MC (2011). Attitudes toward complementary and alternative medicine: a national survey among paediatricians in the Netherlands. Eur J Pediatr.

[14] Posadzki P, Watson LK, Alotaibi A, Ernst E (2013). Prevalence of use of complementary and alternative medicine (CAM) by patients/consumers in the UK: systematic review of surveys. Clin Med (Lond).

[15] Harris PE, Cooper KL, Relton C, Thomas KJ (2012). Prevalence of complementary and alternative medicine (CAM) use by the general population: a systematic review and update. Int J Clin Pract.

[16] Fisher P, Ward A (1994). Complementary medicine in Europe. BMJ.

[17] Patel SJ, Kemper KJ, Kitzmiller JP (2017). Physician perspectives on education, training, and implementation of complementary and alternative medicine. Adv Med Educ Pract.

[18] Jan M, Basamh M, Bahassan O, Jamal-Allail A (2009). The use of complementary and alternative therapies in Western Saudi Arabia. Saudi Med J.

[19] Vlieger AM, van de Putte EM, Hoeksma H (2006). The use of complementary and alternative medicine in children at a general paediatric clinic and parental reasons for use [Article in Dutch]. Ned Tijdschr Geneeskd.

[20] Kemper KJ, Vohra S, Walls R (2008). The use of complementary and alternative medicine in pediatrics. Pediatrics.

[21] Sanders H, Davis MF, Duncan B, Meaney FJ, Haynes J, Barton LL (2003). Use of complementary and alternative medical therapies among children with special health care needs in southern Arizona. Pediatrics.

[22] Sibinga EM, Shindell DL, Casella JF, Duggan AK, Wilson MH (2006). Pediatric patients with sickle cell disease: use of complementary and alternative therapies. J Altern Complement Med.

[23] Hurvitz EA, Leonard C, Ayyangar R, Nelson VS (2003). Complementary and alternative medicine use in families of children with cerebral palsy. Dev Med Child Neurol.

[24] Sikand A, Laken M (1998). Pediatricians' experience with and attitudes toward complementary/alternative medicine. Arch Pediatr Adolesc Med.

[25] Marie S, Almutairi S, Al Turki M, Alsabty N, Almodaimegh H (2018). Primary and specialized physicians’ knowledge of and attitudes towards the use of complementary and alternative medicine in medical practice. Egypt J Hosp Med.

[26] Sawni A, Thomas R (2007). Pediatricians' attitudes, experience and referral patterns regarding complementary/alternative medicine: a national survey. BMC Complement Altern Med.

